# Testimony of Medical Experts*Extracts from an address before the Buffalo Medical Association, April 6, 1880.

**Published:** 1880-05

**Authors:** Charles Beckwith

**Affiliations:** Judge of the Superior Court of Buffalo, N. Y.


THE BUFFALO

MEDICAL AND SURGICAL JOURNAL.

Vol. XIX.—MAY, 1880.—No. 10.

### Original Communications.

### TESTIMONY OF MEDICAL EXPERTS.*

BY HON. CHARLES BECKWITH,

Judge of the Superior Court of Buffalo, N. Y.

First we have the parties, the people on one side and the prisoner on the other in criminal prosecutions, and in civil actions the plaintiff or party who alleges certain facts to be true upon which his rights depend, and the defendant who denies the truth of the allegations. These parties are supposed to be attended in court by counsel learned in those fundamental laws which define their rights or impose obligations, and who are also supposed to be skilled in those minor laws which govern the *processes* of legal investigation, the rules of practice and the principles of evidence. It is the office of the presiding judge, by the aid of the counsel, to take care that the practical steps in the proceeding are taken according to established regulations, and to observe that the proofs adduced are such as are just and fair between the parties, that is to say, are of a character that do not violate the principles of the law of evidence, that is, those

* Extracts from an address before the Buffalo Medical Association, April 6, 1880.

precepts and rules to which I have already referred as having been found out and prescribed by the courts during some centuries of experience as best calculated to subserve the interests of truth and justice. He is to see to it that neither party puts into the balance any unlawful weight, that neither party gets any irrelevant fact or testimony into the problem that is before the court for its solution. And then, when the proofs of the parties are in, it remains for the jury, according to the common explanation, under proper instructions from the judge as to the law applicable to the case, to estimate and weigh the testimony and facts in proof, and from them to deduce the conclusion whether the right asserted by the plaintiff is well founded. The true theory as to the part of the jury is, I think, that the conviction which is produced by the proofs so admitted under the watchfulness of counsel and supervision of the court, upon the minds of twelve fair and impartial men, is for all practical purposes the true solution of the question submitted. For, it may be assumed, that, under fair circumstances, the proofs that bring the minds of twelve men to one and the same conclusion, would convey the minds of mankind generally to the same judgment. Thus it is we are supposed to reach that probability of truth, that conclusion and judgment upon which men may safely act in the practical affairs of life.

Undoubtedly this is an imperfect apparatus, so to speak, and the results obtained are subject to some uncertainty, but surely the imperfection is not greater or the issues more uncertain than those which pertain to human affairs in general. Indeed, I believe that the experience of a long period of years in England and this country has failed to convince our ablest and most experienced jurists that a better plan can be devised for the adjustment of those differences and unfortunate controversies which arise among our fellow men. And I am informed that on the continent in Europe the impression is gaining ground that the system is a better one than that which has prevailed there.

Now, when a legal investigation has been instituted in one of our courts, it is the duty of counsel and the judge to restrain a witness from testifying to any thing that is not within his personal knowledge, for it is a fundamental principle in the law of evidence that a witness may testify only to those matters that are within his own knowledge, and not to those he has learned from other persons or from public rumor. But the witness, it may be, is a stranger to the court and the jury; they know nothing of his personal history, his character, or his relations to the parties litigant, and consequently do not feel quite assured what estimate to put on his testimony. The expedient has been adopted of allowing the opposing counsel to cross-examine the witness, in order that it may be discovered whether he really knows the matters of which he testifies, and to disclose whether he had any interest, or any inducement to color his statements, though they may be literally true as to facts. And so in our system of trials the right of cross-examination has become established, and its exercise is regarded as one of the safest tests of the value of testimony. The interest felt by the party affected adversely by the testimony will sharpen his wits for the detection of any bias, or prejudice, or want of exact information on the part of the witness.

While the general principle is that a witness may testify only to what is within his own knowledge, there is one important exception to the rule. It is this, that men of science or skill, may, under certain conditions, give their *opinions* in evidence. At least lawyers are accustomed to say that they may give in evidence their opinions, though I apprehend that upon investigation and analysis the *opinions*, as such, are not taken as elements of proof, but rather the general facts, laws, principles or inductions to which those opinions relate.

A physician or surgeon, a lawyer, civil engineer or other person skilled in a science or art, may be brought into court as a witness in three different capacities, as it were; first, to testify like the common witness to some incident or ordinary fact that has fallen under his observation. [illegible]ough he may be a man of skill, following a professional avocation, in this case, he stands on a par with his fellow men; he cannot certainly safely decline to obey the summons that calls him into court, and when he has come into court he testifies under the same conditions as the ordinary witness. He is entitled to no other fee or compensation than that allowed other witnesses; he can claim no privilege; neither can he, in such case, be permitted to express his opinion on the facts.

Secondly, he may come into court to testify to those facts belonging to the art or science in which he is skilled, but which facts lie beyond the observation and knowledge of men in general. Though the facts to which he testifies are those which only a person skilled in his profession could verify, still, when proven, they take their place among the data of the case in group with the facts established by the other witnesses.

Thirdly, he may come into court as an expert to give in evidence his *opinion* upon the facts to which he testifies himself, or to which others have given testimony, or upon facts assumed hypothetically for the purpose of taking his opinion.

What is the nature and use of an expert's *opinion* taken upon a trial as evidence? The opinion is not given or received as in any manner advising the court or jury what decision to render. But there may be relations of cause and effect in physical facts, of which it is necessary to make proof; or a conventional regulation the existence of which is necessary to be produced for the consideration of the jury, in order to a just finding, but the processes by which the skilled witness is able to verify the existence of that physical law or conventional necessity, would be beyond the comprehension of an unskilled jury. There, then, the expression of the witness' *opinion* is taken as the compend of all the facts upon which the opinion was formed as well as the conclusion which the jury would have reached if capable of the deduction.

The principal remedy afforded by our rules of practice for the discovery of errors in expert testimony, is the cross examination. It has been claimed with some show of reason that when suitors ask the opinion of professional gentlemen, and require for that purpose their involuntary presence in court, that they should be content with the opinion expressed, without the indignity of inquiring into the grounds thereof and into the extent of the witness' attainments in the branch of knowledge involved. Nevertheless, it is not easy to see how, consistently with the plan of a common law trial, the right of cross-examination can be safely abridged. I have set forth enough of the theory of common law procedure to show the necessity, while trials are conducted according to that system, of reserving to a party the right of cross-examining the witnesses of his adversary. It is a right which must be reserved for the discovery of truth, though it may be but sparingly used, as it is, sparingly and cautiously, by the most skillful and experienced counselors.

This right or privilege no doubt is sometimes shamefully abused, almost beyond the power of the court to prevent it, without depriving the *party* of the right his counsel misuses. It is intolerable to any honorable man's sense of decency to see the insolent manner counsel sometimes put on, and the barren impudence of questions put to witnesses who have been called upon the stand simply because they were unfortunate enough to possess the knowledge of something a party to a law-suit wishes to use. Such conduct seems peculiarly out of place when honorable professional gentlemen, scholars, men of science, are called into court to give the cause of justice, as it is called, the benefit of their researches, their knowledge, their skill.

But every witness called into court may remember that the insolence of a counselor is usually in the inverse proportion of the discipline and cultivation of his mind, and his capacity to try his suit like a lawyer, and that such is the common judgment of the profession. I think a great improvement in this regard is going on among members of the bar. I am glad to see that this matter of the treatment due witnesses, and especially medical experts, has been brought before the State Bar Association, and that the practices complained of have been denounced as unbecoming a member of the bar. A report of a committee of that Association declares that "it is cowardly to attack unfairly a person to whom the court allows no reply." Public opinion, which controls every thing, is working a change in the manners and modes of courts and lawyers.

Not many years ago, not infrequently, a trial was less a trial between the parties to the suit, than a contest for superiority between lawyers, a struggle of legal athletes for mastery, and the prize of popular applause, and when the pulse and sympathy of the crowd, felt of course by the jury, betokened or announced which of the two athletes was victor, the cause of the client was too often won or lost accordingly.

I would not attempt before this learned society to reflect any light upon those questions of science which medical men are most frequently called upon to elucidate in court, nor to say what modes of exposition are best adapted to those occasions. But the observance of one or two suggestions will certainly tend to preserve the witness from distasteful contests with counsel. I will venture the preliminary remark that the reputation of a physician or surgeon is seldom hurt or advanced by any thing that occurs in a court-room, unless it may be among members of his own profession. The fate of the prisoner, or the issue of the cause, enlists the spectators, and the peculiar discipline of lawyers will cause them rather to respect most those opinions that are advanced with reserve and caution. *Curia advisari vult* expresses the judicial mode, that upon important matters the court will take time to consider and consult. The like caution on the part of a medical expert in advancing an opinion in a court of justice, I am quite certain will not weaken the force of his statement, or the respect it receives at least among lawyers. And I see no reason why the suggestions offered by Professor White before your society on a former occasion were not sound—that a medical expert need not hesitate to say to a question propounded "I do not know;" or to declare that upon the facts presented or data given he cannot express an opinion, cannot undertake to state the proper inference.

It is certain that in the courts the assumption of familiarity with every thing belonging to the expert's branch of science, does not give weight to this testimony. In a criminal cause in this state, in which a number of eminent medical experts had testified on either side of the case, the court referring to the subject of what degree of credence should be given to the opinions of medical experts generally, said: "They have their theories and speculations, and the difficulty with them many times seems to be that they are hardly willing to admit that there is much in the human system, its ailments and diseases that is beyond their knowledge and comprehension."

An expert witness on the stand will make his examination comparatively pleasant by listening carefully to the question put to him by counsel, and when the question is understood, by responding to it with extreme simplicity of statement, but with that fair fullness which is the measure of candor, yet answering neither more or less than what is called for by the question. For, otherwise, if he volunteer, he will excite the jealous watchfulness of opposing counsel, provoke his onslaught, and debar himself from the protection of the court. For the *opinion* of a medical expert is to be given on the facts proven, or the hypothetical facts assumed for the purpose of taking his opinion thereon. If it appear from the answer that the witness has assumed or taken into the proposition a fact not given in the question, the utility of the answer is lost, for the court will not suffer an answer that is not responsive to the inquiry, to go to the jury. It is not the business of the expert to correct the formulae of counsel. If the expert sees that the counsel, from ignorance or otherwise, has omitted facts, or omitted data from the hypothetical case stated, in the absence of which no proper conclusion can be drawn, no opinion can be stated, his safer course is to say that he cannot answer the question as put. If the lawyer who acts as counsel is not able to collate the proper facts or data for the opinion, let him go without his answer. The court, however, on application will usually allow the witness to explain why he cannot answer the question in the form presented.—*Concluded in June number.*

